# Supplementary material for: Computational synthesis of locomotive soft robots by topology optimization
Source: Sci Adv. 2024 Jul 24;10(30):eadn6129. doi: 10.1126/sciadv.adn6129 (PMC11268422; doi:10.1126/sciadv.adn6129)
Supplement: Supplementary file 1 — Sections S1 to S4 Table S1 Figs. S1 to S9 Legend for data S1 Legends for movies S1 to S9 [file sciadv.adn6129_sm.pdf]

Science Advances
AAAS

# Supplementary Materials for

## Computational synthesis of locomotive soft robots by topology optimization

Hiroki Kobayashi *et al.*

Corresponding author: Tsuyoshi Nomura, nomu2@mosk.tytlabs.co.jp

*Sci. Adv.* **10**, eadn6129 (2024)
DOI: 10.1126/sciadv.adn6129

**The PDF file includes:**

Sections S1 to S4
Table S1
Figs. S1 to S9
Legend for data S1
Legends for movies S1 to S9

**Other Supplementary Material for this manuscript includes the following:**

Data S1
Movies S1 to S9

**S1 Mass distribution change in walker optimization**

Fig. S3 shows the changes in the robot's center of gravity and mass during optimization. Fig. S3(a) shows the center of gravity history overlaid on the optimized shape image. Fig. S3(b) shows the plot of the center of gravity and the mass through the optimization iteration. The mass increased rapidly in the early stage of optimization and then decreased gradually in the latter half. In the initial iterations, the center of gravity was raised vertically because the lower structure was softened to move the leg-like structures. Then, it was lowered back by removing unnecessary material from the upper part. Furthermore, the center of gravity moves continuously forward throughout the optimization and remains steady at the rear end of the front leg, which is suitable for retracting the back leg after stepping out.

**S2 Effect of Young's modulus and pressure settings on locomotion**

To investigate how Young's modulus and pressure setting affects the soft robot locomotion, we have performed simulations for double Young's modulus with original pressure, and double Young's modulus with double pressure, compared with original properties (Fig. S4). The shape of soft robot is the same as *walker*, to compare the effect of modulus and pressure only. In double Young's modulus with the original pressure, the stride of soft robot was shorter (7.49 mm) than that of the original modulus (9.64 mm). On the other hand, the stride became longer (12.5 mm) in double Young's modulus and double pressure.

However, the increased stride did not effectively improve the locomotion distance. As shown in Fig. S5, the locomotion distance, which is the center of gravity change in X-axis, is 41.2 mm in double modulus and double pressure case. Despite a 30% increase in stride length, the locomotion distance improvement remains less than 8%. This can be attributed to an increase in bounce and a deviation from the original walking behavior, as can be seen from the position of the center of gravity in the Z-axis direction. These discussions suggest the material coefficients and pressure have a substantial impact on the optimal design of soft robots, and optimal shapes of soft robots vary depending on the change of material modulus or pressure.

**S3 Experiment for intermediate iteration design in optimization**

The intermediate designs at the 20th and 100th optimization iterations were fabricated with the same material and print setting as that for the optimized design. The 3D models were extracted from isosurfaces at 50% density ($\gamma = 0.5$). Note that the isolated part at the bottom in 20th iteration design, which is shown in Fig. 3A, has been removed for 3D printing. The pressure and its frequency are set to 50 kPa and 5 Hz with 50:50 duty ratio, same as the setting for the optimized design.

Fig. S6 shows the experimental results of the 20th and 100th iteration designs. Compared to the optimized design (Fig. 2E), the locomotion speeds of the intermediate designs were noticeably shorter, i.e., 6.42 mm/s at the 20th iteration design, and 22.4 mm/s at the 100th iteration design. The trend of locomotion distance is consistent with the increase of objective function as optimization proceeds. In the early stages of optimization, more material is used to make the structure viable. As a result, the strides are shorter due to smaller deformations. These results confirm that updating the design in optimization calculations contributes to improved performance in reality.

**S4 Mass distribution change in climber optimization**

Fig. S7 shows the changes in the robot's center of gravity and mass over the whole iterations. Fig. S7(a) shows the center of gravity history overlaid on the optimized shape image. Fig. S7(b) shows the plot of the center of gravity and the mass through the optimization iteration. Similar to the *walker*, the mass of the *climber* increased early in the optimization and then decreased. The mass of the final design is lower than that of *walker* (Fig. S3) because *climber* locomotes against gravity. The center of gravity moved to the front part, which required the complex action of extending the

arms forward and then holding the arms against the wall, but as the wasted material was removed, it returned to near the center of the chamber.

**Table S1. Estimated relative moduli $g_\infty$, $g_i$ and corresponding relaxation time $\tau_i$ in Prony series.**

| Number of Maxwell elements | $g$ (-) | $\tau$ (s) |
|---|---|---|
| ∞ | $9.06\times10^{-4}$ | - |
| 1 | $6.36\times10^{-4}$ | $2.73\times10^{-1}$ |
| 2 | $2.09\times10^{-3}$ | $7.56\times10^{-3}$ |
| 3 | $1.27\times10^{-2}$ | $2.09\times10^{-4}$ |
| 4 | $1.25\times10^{-1}$ | $5.77\times10^{-6}$ |
| 5 | $8.59\times10^{-1}$ | $1.59\times10^{-7}$ |

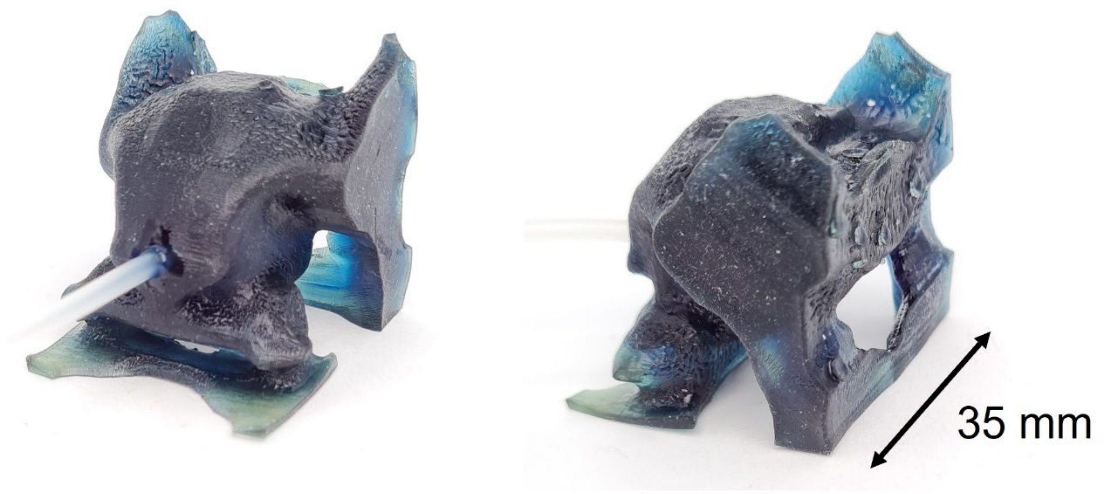


**Fig. S1. Prototype of soft robot used for the air pressure measurement.** This prototype consists of the materials described in “Soft robot fabrication” section.

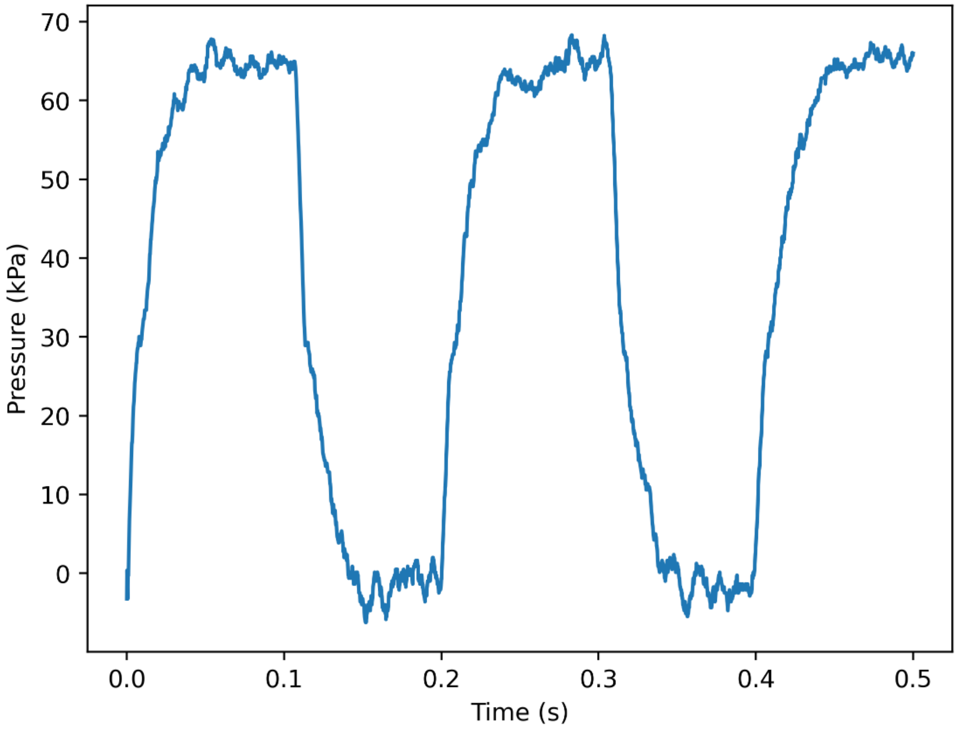


**Fig. S2. Waveform of the pressure used for the simulation.** The pressure was measured for the prototype shown in Fig. S1. The data is available in Data S1.

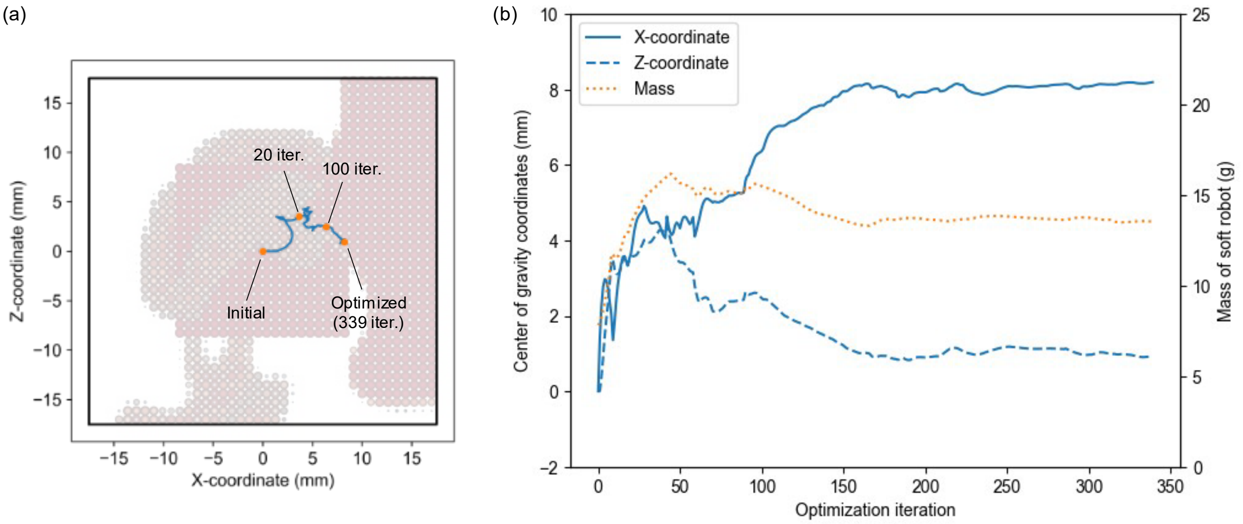


**Fig. S3. Center of gravity coordinates in static state and mass of the robot in *walker* optimization.** (a) The center of gravity coordinate was changed through the design updates. The plot is overlaid on the optimized shape image. The coordinate origin is defined as the center of pneumatic actuator. X-axis and Z-axis are walking direction and vertical direction, respectively. (b) Detailed plot of the robot's center of gravity and mass. The center of gravity moved forward and slightly upward as the optimization proceeded. The mass was increased in early stage of optimization but decreased in the later iterations.

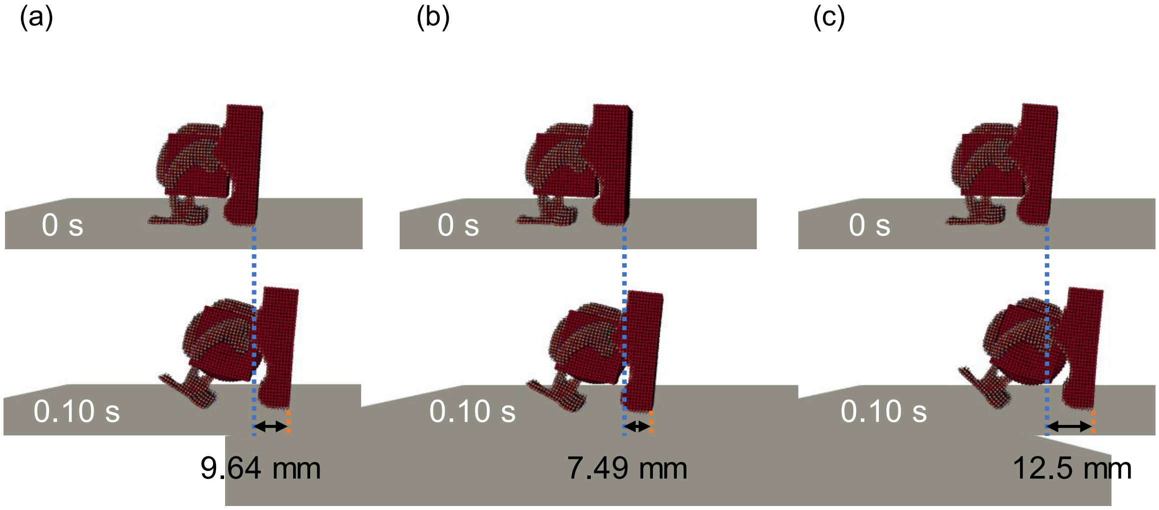


**Fig. S4. Stride distances for different Young's modulus and different pressure settings.** (a) original modulus and pressure. (b) double modulus with original pressure. (c) double modulus with double pressure.

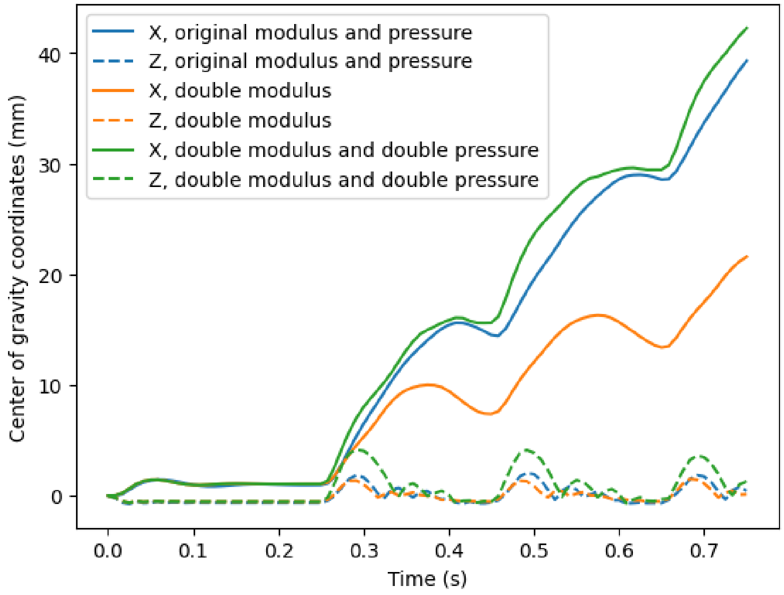


**Fig. S5. Center of gravity plot for different Young's modulus and different pressure settings for *walker*.** X-axis is the walking direction (horizontal), and Z-axis is the vertical direction. The origin of center of gravity is based on initial state (0 s).

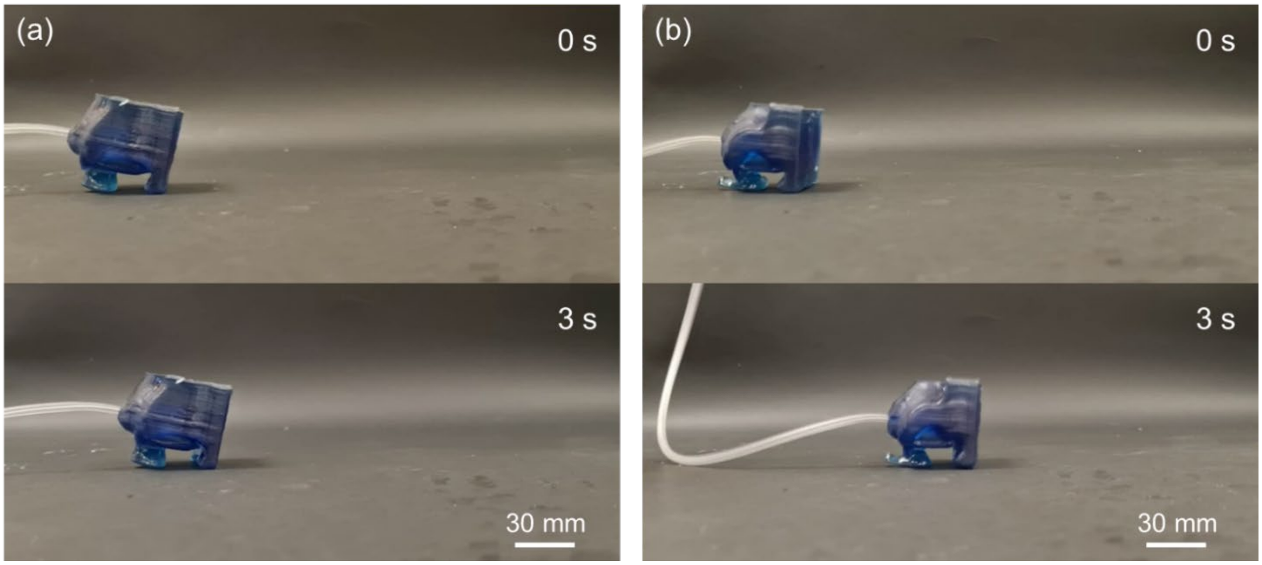


**Fig. S6. Experimental results of intermediate design for walking task in 20th and 100th optimization iteration.** The soft robots are fabricated based on isosurfaces of 50% density ($\gamma = 0.5$). (a) Snapshots of locomotion in 20th iteration design. The locomotion speed was 6.42 mm/s. (b) Snapshots of locomotion in 100th iteration design. The locomotion speed was 22.4 mm/s.

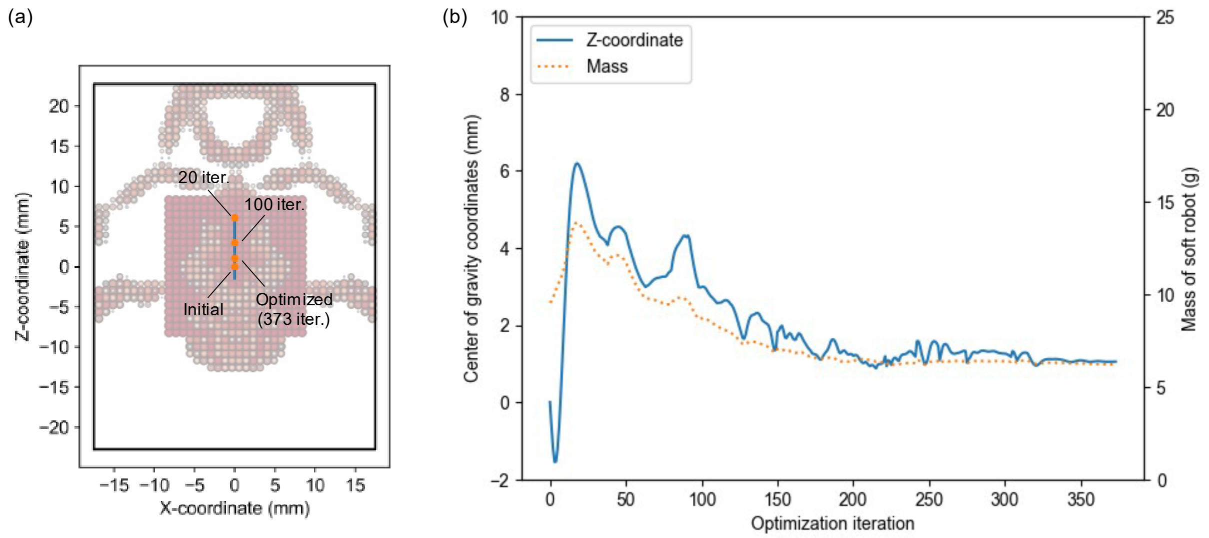


**Fig. S7. Center of gravity coordinates in static state and mass of soft robots in the *climber* optimization.** (a) The center of gravity coordinate was changed through the design updates. The plot is overlaid on the optimized shape image. The coordinate origin is defined as the center of pneumatic actuator. Z-axis is climbing direction. (b) Detailed plot of the robot's center of gravity and mass. The center of gravity moved upward. The mass was increased in early stage of optimization but decreased in the later iterations.

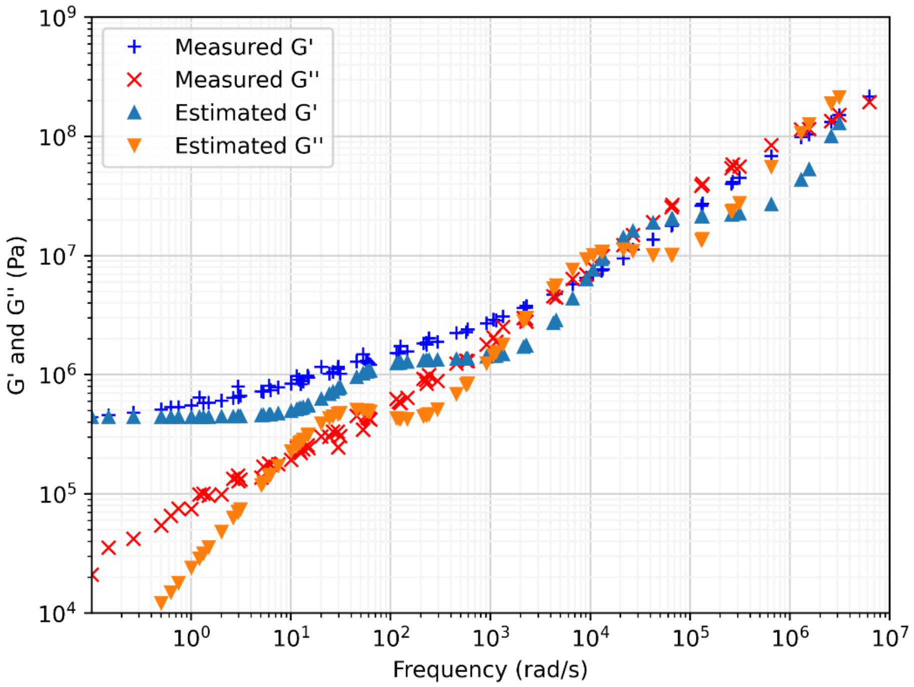


**Fig. S8. Frequency response diagrams of measurement results by DMA and estimation results using three Maxwell elements.** *G*' and *G*'' represent the storage modulus and the loss modulus, respectively.

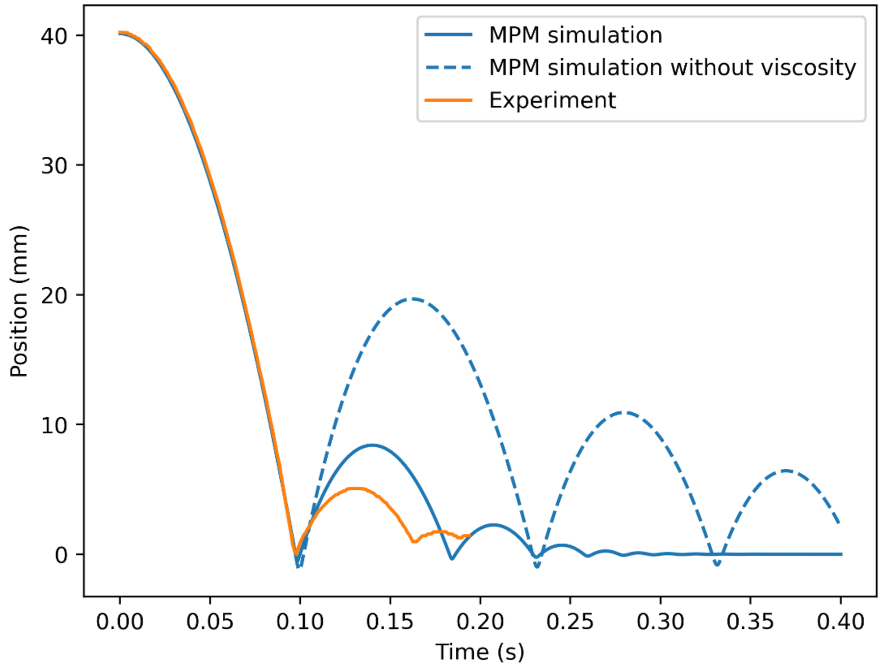


**Fig. S9. Time-series coordinate in vertical direction for the ball drop test.** To verify the effect of introducing viscosity, the MPM simulation result without viscosity are also shown.

**Legend for Data:**
Data S1.
Pressure waveform measurement data used for simulation.

**Legends for Supplementary Movies:**
Movie S1.
Shape synthesis process of *walker* and locomotion behavior over several optimization iterations.
Movie S2.
Comparison of *walker* locomotion between MPM simulation and experiment.
Movie S3.
*Walker* locomotion at 0%, 10%, 20%, and 30% inclines.
Movie S4
Locomotion of 20th and 100th iteration design for walking task.
Movie S5.
Optimized results in walking task on 30% and 57.7% (30-degree) inclines.
Movie S6.
Shape synthesis process of *climber* and locomotion behavior over several optimization iterations.
Movie S7.
*Climber* experiment result at 5 Hz.
Movie S8.
Comparison of *climber* locomotion between MPM simulation and experiment.
Movie S9.
*Climber* experiment for an extended time.
